# Commanding with compassion: harnessing the potential of military chaplains within the NATO structure

**DOI:** 10.3389/fpsyt.2025.1599662

**Published:** 2025-05-30

**Authors:** Jan Grimell, Tatiana Letovaltseva, Janne Aalto, Hans De Ceuster

**Affiliations:** ^1^ Department of Sociology, Faculty of Social Sciences, Umea University, Umeå, Sweden; ^2^ Faculty of Theology and Religious Studies, Katholieke Universiteit (KU) Leuven, Leuven, Belgium; ^3^ Finnish Defence Research Agency, Finnish Defence Forces, Helsinki, Uusimaa, Finland; ^4^ Humanist Chaplaincy, Belgian Armed Forces, Brussels, Belgium

**Keywords:** military chaplain, NATO, pastor, priest, humanist, support, military spiritual care, military pastoral care

## Abstract

The purpose of this article is to foster a better general understanding of military chaplains within NATO by elucidating their roles, highlighting what distinguishes military chaplains from military medical (psychiatrists and psychologists), and offering general suggestions on how military commanders at the tactical (battalion) level can benefit from military chaplains. The context for this paper is the ongoing research conducted by NATO’s Science and Technology Organization (STO) Human Factors and Medicine (HFM) Research Task Group (RTG) 352, titled “Moral Challenges in the Future Security Environment (FSE): Guidance for Leaders”, which began in 2022 and will continue through 2025. The research methodology employed in this article is known as collaborative inquiry, which emphasizes a partnership between academic researchers in military chaplaincy and practitioners (i.e. military chaplains) with the aim of bridging the gap between theory and practice. The following three questions have guided the collaborative methodology: What do military chaplains typically do when supporting service members? What concepts are used to describe their work? What distinguishes military chaplains from medical professionals? The results are presented in the article and describe, among other things, that despite significant variations among NATO members’ military chaplaincy services, chaplains generally exercise a *ministry of presence*. They are trained to address a wide range of spiritual, religious, moral, ethical and existential issues among service members and veterans. Additionally, non-clinical concepts related to moral injury (not yet a clinical diagnosis), such as guilt, shame, forgiveness, reconciliation, fall within the expertise of chaplains. Chaplains’ commitment to professional secrecy and confidentiality – considered absolute by some chaplains – as well as the ethical distance (from the command structure) provides a safe space for military personnel and veterans to express their feelings, thoughts, and experiences. Additionally, military chaplains are often experts in dealing with death and grief, enabling them to conduct ritually and morally dignified farewells for fallen soldiers and provide support to their comrades and units. These are just some examples of the areas of expertise that military chaplains typically master.

## Introduction

One of the significant discussions both within the military services and outside them is the understanding of the roles of military chaplains. It has been reported that military chaplains engage in a variety of compassionate activities to demonstrate their presence and practice contemplative listening while performing their duties (1). Killing, death, suffering, grief, anxiety, and anguish are inherent aspects of military training, deployment, and combat (2–5). Ontologically, the military profession involves mastering and managing killing and death, which ultimately influences how service members are affected both individually and collectively (3, 6, 7). The combat stress, post-traumatic stress, and aftermath of death have been addressed clinically through the theories, methods, and evidence-based practices of military psychiatry, especially since its significant advancement during the two world wars of the 20th century (1, 8). Despite the prominence and dominance of military psychiatry (1), military chaplains remain a permanent element within NATO member armed forces. Military chaplains, who have historically been known as priests, pastors, and religious leaders, predate the era of military psychiatry and have existed in some armed forces for many centuries (2).

Historically, religious representatives (i.e., priests, pastors, and religious leaders) have played a crucial role in providing theological and moral justification for waging war or engaging in righteous warfare against perceived enemies (9, 10). As secularization led to a clearer separation between state and religion/church, this role has diminished, though discussions of morally justified wars persist (3). However, the significance of military chaplains in armed forces has not decreased due to secularization. On the contrary, military chaplains have become more relevant, given the increasing research and interest in military chaplaincy, particularly concerning morality and moral injury in warfare (2, 3, 5, 10–16). One reason for the continued presence of military chaplains is that war is an ancient, timeless social phenomenon that raises profound existential questions, which warriors have grappled with throughout history (2, 17). Military chaplains specialize in addressing these existential, ethical and moral issues (12, 18, 19).

Another rationale for the presence of military chaplains is the legal right and need for service members with religious and spiritual identities to practice their beliefs during military service and deployment, especially in difficult moments, as these identities provide support, meaning, and direction. This necessity aligns with the pluralization of belief systems and faith communities in a late or postmodern context (20, 21). In several nations, the pluralization and secularization of society have paved the way for the introduction of humanist chaplains to meet the needs of soldiers who do not identify with any religion. These chaplains provide conversational support, ethical guidance, and help with existential issues without a religious foundation. It reflects an increased respect for diversity and freedom of religion within the military and recognizes the spiritual needs of non-religious soldiers. The goal is to ensure that all soldiers have access to support, regardless of their faith or worldview (22–24).

Military chaplains are present in the armed forces of almost all NATO countries, reflecting each nation’s ecclesiastical, religious, spiritual, and humanist traditions (23). As a transnational defense organization, NATO does not regulate military chaplaincy services as a unified resource; each country organizes its chaplaincy according to its own traditions, cultures, and laws. While military chaplaincy is a common feature within NATO, the organization and utilization of military chaplains (e.g., role, duty, function, representation of faith communities, training, requirements, employment, chain of command, etc.) vary widely among member countries, influenced by each nation’s history, traditions, culture, and legal framework. Thus, it is challenging to make broad generalizations about the structure, purpose, and functions of military chaplaincy services across NATO, and this can only be achieved to a limited extent.

However, it is crucial to understand how to maximize the use of military chaplains in addressing the moral challenges of future security environments and facilitating joint operations between NATO allies by leveraging chaplaincy resources through interoperability.

The moral challenges in particular stand out as especially significant and have been clearly described by the NATO Science and Technology Organization (25), in the activity details for the Human Factors and Medicine Research Task Group 352, as an important area of research that is not yet well understood:

“Military operations have always involved moral challenges, as they implicate fundamental values (right vs. wrong, just vs. unjust) and affect the well-being of others. These moral challenges are often faced under a range of powerful operational stressors (e.g., risk, time pressure, sleep deprivation), which can increase the difficulty of responding appropriately. Moreover, although militaries are focused on exploiting new technologies and capabilities, the implications of these for operational ethics and the related psychological outcomes for personnel are not well understood.”

This paper attempts to provide such an understanding of military chaplains within the NATO structure.

## Purpose, research questions, and methodology

The context for this paper is the ongoing research by NATO’s Science and Technology Organization (STO) Human Factors and Medicine (HFM) Research Task Group (RTG) 352 on “Moral Challenges in the Future Security Environment (FSE): Guidance for Leaders.” HFM-RTG-352 began in 2022 and will continue until 2025 (see NATO Science and Technology Organization activity details, 2022). The research group has identified military chaplains as an important category within the NATO structure with a unique capacity to address moral challenges. Although there is a considerable body of national-level research on military chaplaincy within NATO countries, there is a notable scarcity of articles providing a generic understanding of the role of military chaplains. This gap serves as the rationale for this article.

The topics presented in this article were formulated during the group’s collaborative inquiry into a number of research questions. This particular research involved a subset of the group, including researchers and military chaplains, who are focused on military chaplaincy.

The purpose of this article is to foster a better general understanding of military chaplains within NATO by elucidating their roles, highlighting what distinguishes military chaplains from military medical professionals (psychiatrists and psychologists) and offering general suggestions on how military commanders at the tactical (battalion) level can benefit from military chaplains.

The following research questions have been derived from the purpose:

1. What do military chaplains typically do when supporting service members?
2. What concepts are used to describe their work?
3. What distinguishes military chaplains from medical professionals?

The answers to these research questions form the foundation for discussing and suggesting ways to unlock the potential of military chaplains. This can help in the referral system within the military services for commanders enhance the sustainable development goal of good health and wellbeing of the military members. Understanding the role of military chaplains is crucial for commanders to maximize their benefits during pre-deployment, deployment, and post-deployment. Additionally, it is important to understand and recognize the limitations, particularly concerning the interoperability among military chaplains from different NATO countries and the diverse chaplaincy needs of service members from various faith and philosophical traditions in multinational units.

In addition to offering suggestions to commanders, it is important to recognize that other support functions within the armed forces and NATO, as well as the research community, may have a broader interest in understanding military chaplains. Therefore, this article is aimed at an audience that may not be very familiar with the subject but seeks to understand the cross-national features, tendencies and contributions, concepts, distinctions, and potential of military chaplains.

The research methodology used in developing this article is known as collaborative inquiry (26). It emphasizes the partnership between academic researchers in military chaplaincy and practitioners, i.e., military chaplains in the field, aiming to bridge the gap between theory and practice. The methodology of a collaborative inquiry involves a group of researchers and practitioners working together to explore a specific issue or question (27). The group collectively reflects on their practices, generates knowledge, and applies it to improve practice (28). Since HMF-RTG-352 began in 2022, participants have engaged in systematic reflection and discussion for one working week per semester. These meetings alternate between participating countries. So far, they have taken place in France, Belgium, Canada, the United Kingdom, and the USA. One final meeting is planned in Sweden in spring 2025, followed by a preliminary closing meeting in Canada in the fall, pending NATO approval. In addition to in-person sessions, participants also hold online meetings.

### Data collection

During the collaborative sessions, discussions and reflections were documented through personal notes and group meeting notes. Between meetings, participants gathered and collected various types of research-related or practice-based information, which was then presented and discussed during the sessions. Presentations, shared documents, collaborative group documents, internal workflows, task allocations, and internal working guidelines are available but are not classified as public releases.

The literature employed in the article includes peer-reviewed articles and books by scholars from theology, practical theology, spiritual care, sociology, medical sociology, sociology of religion, military medicine (psychiatry and psychology included), all relevant to the topic of military chaplaincy. The keywords that guided the search and selection of literature were: military chaplains, chaplains, pastoral care and counseling, spiritual care and counseling.

### Data analysis techniques

Typical of the collaborative inquiry is that the group (composed of researchers and practitioners) collectively reflects on their practices, generates knowledge, and applies it to improve those practices—an approach that in itself represents the methodology (27, 28), along with the tasks we have worked on over the years. The collaborative analysis has involved discussing presentations, research findings, and professional practices in order to address the three questions posed in this article, which has, in turn, been a significant contribution to the group’s overall work. This collective body of material has been interpreted through the lenses of both researchers and practitioners and has been synthesized and thematized in the responses to the research questions developed in the article.

### What do military chaplains typically do when they support service members?

Answering the question of what military chaplains do when they pastor, support, and assist service members, units, leaders, and, in some cases, military families is challenging because their roles can vary significantly across different armed forces (e.g., 5, 19, 29–32). Drawing on insights gained through collaborative inquiry with academic researchers and military chaplains, the responsibilities and actions of military chaplains depend on their specific purposes, levels of integration and independence within command structures, mandates, backgrounds, and education—all of which shape their identities and missions. Additionally, what military chaplains can do is influenced by societal laws, military regulations and policies, and the principles and rules of the faith communities they represent (29).

The context of their efforts—whether during basic training at a home base, in a pre-deployment phase elsewhere, during deployment in conflict or war zones, or in the post-deployment phase at home—also significantly impacts their activities. For a full description of the characteristics and challenges of deployment phases, see STO Technical Report TR-HFM-329 (2025) A Psychological Guide for Leaders Across the Deployment Cycle (33). These different situations create varying conditions that military chaplains must adapt to, limiting or expanding their scope of activities. For example, the role of a chaplain at a home base, where they can move relatively freely and potentially support military families, is different from that of a chaplain deployed on a ship or in a war zone, where some of them must stay within the camp. These situational aspects also affect the existential, spiritual, ethical and moral issues that military chaplains encounter on individual (e.g., a service member’s conflicting feelings about purpose, morality, and behavior) and group/organizational levels (e.g., advice, lessons, lectures, classes, ceremonies, rituals, care, and support related to death, loss, and grief).

In some countries, the number of military chaplains is insufficient to address all related needs. Consequently, inter-professional collaboration is sometimes necessary. However, this can lead to confusion among military personnel and authorities regarding the competencies, mandates, and ethical and legal frameworks of different caregivers. Therefore, it is essential to clarify the identity, competencies, and ethical and legal frameworks of each professional, including chaplains. Recently, chaplaincy interoperability initiatives, such as the NATO Navy Spiritual Support Interoperability concept, the NATO and partners chaplains’ operations course, and the NATO’s Science and Technology Organization Human Factor and Medicine’s Exploratory Team 209^1^ on the spiritual dimension of military medicine, have begun exploring the complexities of this topic to find viable interoperable ways forward. Adopting a salutogenic and holistic approach to health and well-being, the contribution of chaplains can be a significant asset, particularly in mental health (34).

Military chaplains are experts in addressing existential, religious, philosophical, ethical, moral and spiritual questions, both broad and specific (1, 12, 19, 35, 36). These questions can pertain to the meaning and purpose of life, identity, death, grief, evil, ethics, and morality. The connection with ethics and morality is also significant when addressing moral injury in non-clinical ways, as managing related concepts such as guilt and shame is a key aspect of the military chaplaincy profession (36). They may also involve specific religious issues or matters with a direct or indirect connection to a transcendent dimension, higher power, or God (15, 19, 35, 37, 38). When such topics are addressed to military chaplains, they use their respective “toolboxes”—the theological, philosophical, religious, spiritual, and existential education and training they have received. This includes all the practices, rituals, and ceremonies embedded in their faith traditions. Therefore, how military chaplains tackle these questions can vary greatly between different chaplains and armed forces within NATO.

### The ministry of presence

A common approach among many, if not all, military chaplains is the practice of a *ministry of presence* (39–43; also see 44, 45). This means being present in the lives of service members, willing to listen and support them through their questions, thoughts, hardships, and sufferings. Military chaplains are usually trained to be good listeners, providing support, interpretation, encouragement, and carrying service members’ stories, feelings, thoughts, and struggles with a deep commitment to confidentiality. This confidentiality is a crucial aspect of their ministry, ensuring that everything shared with a chaplain remains private. This is not necessarily the case with other professionals in the armed forces, who may have mandatory reporting duties or participate in various command risk assessments, potentially impacting a service member’s career or reputation. This secrecy is of paramount significance, possibly even critically so, due to the duty of confidentiality upheld by chaplains (46).

The concept of *ministry of presence* also encompasses the idea that faith and humanist traditions and their respective communities, represented by chaplains, are embedded within the armed forces to provide support and assistance to ‘their’ service members and military families (39, 41). This includes instances where chaplains’ activities extend to supporting military families as well.

### Military cultural competence

Military chaplains typically possess a cultural competence within the military context (1, 17, 19, 29) and sometimes even hold military ranks. This cultural competence, combined with the ministry of presence, allows chaplains to conduct their ministry close to service members, understanding military values, meanings, and practices, including wearing the uniform. Their presence in the military context helps them gain invaluable cultural understanding and competence, fostering trust among service members (29). When trust is established, service members are more likely to seek support, aid, and guidance from military chaplains as existential, religious, and spiritual dialogue partners.

### Navigating diversity and promoting social unity

Within the concept of the ministry of presence, a variety of practical and formal tasks exist (e.g., pastoral and spiritual care, education, ceremonies, advisory roles, support to commanders, crisis support, etc.). These tasks vary by nation, reflecting how NATO countries have structured their military chaplaincy services in accordance with their history, ecclesiastical and religious traditions, cultural practices, and civil and military laws and governing principles. This may mean that some military chaplains perform tasks that others do not. For example, in some armed forces, chaplains are part of the chain of command and, in the Nordic context, they may even carry weapons during deployments and participate in lighter missions (47), while in others they do not. Specific questions about the tasks of military chaplains in different countries’ armed forces (and branches) can be answered by the chief of chaplains or other appointed officials.

Chaplains can also foster hope, meaning, and purpose by organizing celebrations, moments of reflection, and inspiring members of the armed forces in their moral and spiritual growth.

By facilitating interfaith dialogue, chaplains and their inclusive ministry can play a vital role in navigating faith diversity within the military setting and bolstering social unity. Leveraging their expertise in various belief systems, they enhance cultural understanding and awareness. They may also act as interpreters—albeit not without controversy—regarding the religious landscape beyond the military context, within a religious operational environment (48).

As legitimate religious and philosophical authorities, chaplains are strategically positioned to detect signs of radicalization among warriors and mitigate the escalation of extreme warrior cultures. Their role encompasses preserving the humanity of warriors and stewarding the spiritual dimension within the realm of warfare (49). Additionally, they may foster connectivity and mutual comprehension between civilian society and the military (50, 51), as well as among diverse cultural communities within NATO nations.

## Military pastoral, spiritual, and existential care

There are several established concepts for describing the care and counseling chaplains provide in various contexts—such as hospitals, hospices, prisons, schools, and police departments—that can be adapted to the military context by adding the prefix ‘military.’ However, these concepts are not universal and may not be applicable to all faith traditions or armed forces, each of which may have its own unique terms and concepts.

### Military pastoral care and counseling

The first concept is pastoral care and counseling, a narrower term typically used within faith traditions and communities that have priests and pastors (ordained or otherwise) (46, 52, 53). These traditions often practice military pastoral care rooted in different church traditions, tailored to specific theologies, church orders, and ecclesial principles (e.g., Protestant, Catholic, Orthodox). The concept of military pastoral care can be applied in military contexts where the ministry of presence aligns with how various church traditions educate and train their priests and pastors for pastoral care and counseling in general ministry (54).

In addition to ecclesiastical education and training, military chaplains often undergo specific military chaplaincy training relevant to their roles and missions in the military context. These requirements are typically determined by the chief of chaplains within the armed forces.

### Military spiritual care and counseling

The second concept is spiritual care and counseling, which is broader and more inclusive. This concept can encompass a wide range of faith traditions and communities, including spiritual or religious leaders or appointed representatives (14, 55, 56). Adding to the complexity, it’s worth noting that priests and pastors are capable of providing spiritual care and counseling as well, indicating a more expansive and inclusive approach compared to what might be implied by terms like ‘pastoral care,’ as suggested by Grimell and Bradby (20). In the military context, military spiritual care involves caring for, counseling, and supporting service members, veterans, and units through military chaplains, whether they are religious leaders or appointed representatives, ordained or otherwise. Military spiritual care is an internationally recognized concept (2, 5, 14, 56, 57).

The concept of military spiritual care is broad enough to encompass a wide range of spiritual perspectives and caregivers. It can be tailored to open and inclusive definitions of spirituality, such as the search for meaning and significance in life (58, 59) and aligns well with concepts like *military* sp*iritual fitness* (60). More theologically open definitions connect spirituality to a divine or transcendent dimension in lived religion (61, 62). The humanist tradition encompasses an atheist spirituality characterized by a profound sense of purpose and meaning, as well as a horizontal connection that extends to all beings within the universe, fostering a deep bond with humanity, all living creatures, and the cosmos at large (63).

Definitions of spirituality are contested and debated, as the concept is shared by theological disciplines, church and religious traditions with foundations in God, the Trinity, the Spirit, Allah, traditions with multiple deities or higher powers, secular and humanist contexts that also has a need to define spirituality in their practices, such as in healthcare (64) and new spiritual movements (65). This diversity can lead to incompatible even competing definitions and approaches among practitioners, spiritual representatives, and researchers from various academic disciplines. In other words, what everyone can agree upon regarding the definition of spirituality is not neither given nor unison.

### Military existential care and support

Depending on the definition of spirituality, there may be a need to consider care from an existential approach, referred to as existential care, in cases where religion and spirituality do not easily fit into the concepts of pastoral or spiritual care and counseling (11). Alternatively, a combination of existential and spiritual concepts, termed “existential spiritual” (66), might be considered.

A term such as existential care or spiritual-existential care is not an established concept but rather presents an opportunity to explore additional ways of conceptualizing military chaplain care approaches in contexts where traditional concepts do not necessarily reflect cultural context and chaplaincy practice. At the same time, it should be noted that the thoughts and issues service members highlight in pastoral and spiritual care often have existential significance. Consequently, existential care and support can function as a generic concept, especially sensitive to the cultural climate of Northern Europe and Scandinavia. This term can describe all the activities military chaplains engage in when discussing life questions and issues with service members in the ministry of presence. Additionally, in some armed forces, military chaplains are increasingly responsible for teaching military ethics and linking it to the overall fitness of soldiers. This connection is crucial when addressing the existential and moral issues of individuals or groups (67, 68).

For the sake of clarity, it should be stated that while *pastoral* and *spiritual care* represent distinct and well-established concepts, *existential care*, according to us, rather reflects a lens for understanding certain aspects of chaplaincy than a clearly defined and widely established concept.

## Distinguishing military chaplains from military psychiatrists and psychologists

A crucial yet not absolute distinction exists between military chaplains and military psychiatrists and psychologists.

### Ontological and epistemological differences

Generally, military chaplains and professionals in military psychiatry and psychology operate within entirely different scientific paradigms regarding ontology and epistemology (1). Military chaplains represent the religious and spiritual realm, anchoring their understanding in an ontology that presupposes a transcendent lifeworld. Epistemologically, their knowledge is derived from holy scriptures, wisdom and philosophical literature, and sacred traditions and practices (11). Their education, training, understanding, and experience are rooted in and interpreted through these ontologies and epistemologies.

In contrast, the psychiatric paradigm (encompassing psychology) is grounded in a wholly different ontological and epistemological framework (1). Psychiatry and psychology advance their knowledge through the randomized selection of participants and controlled testing of hypotheses and research questions (8). ‘True’ knowledge is forged through rigorous research designs that control for various factors that could potentially influence research outcomes (69). There are clear regulations, principles, and research ethics governed by national laws and regulations that control knowledge production in this evidence-based research paradigm. Ontologically, this paradigm relies on empirically demonstrated and known information from controlled and randomized tests, which can be claimed as ‘true’ and thus underpin knowledge production. The emergence of the medical and psychiatric research paradigm is also intertwined with expert interests, power dynamics, and commercial interests, topics that sociology and medical sociology often closely scrutinize (8, 70).

### Historical context and dominance

The two world wars in the 20th century significantly contributed to the advancement of modern medicine, including the pivotal role of psychiatry in classifying and understanding health and illness, especially within military contexts (8, 71, 72). Military psychiatry experienced significant development during these wars, solidifying its position within armed forces globally. This influence extended far beyond the military, shaping the psychiatric field at large (1, 8). Moss and Prince (1) carefully presented this development in a post-structuralist approach, highlighting two major processes within a military context: the military’s imperative need for functional military personnel during wartime and psychiatry’s emerging role in researching and defining what constitutes ‘healthy’ or ‘sick’ individuals. Today, military psychiatry stands as the leading and defining knowledge paradigm and practice within Western military contexts, encompassing recruitment, selection, training, and the screening, evaluation, diagnosis, and treatment of illness (1).

Despite the dominance of military psychiatry in defining health and illness within Western military organizations and among veterans (1, 73), military chaplains continue to play a crucial role, given the existential, moral and spiritual complexities of war, within military organizations and among veterans worldwide (5, 12, 14, 15, 29, 47, 62, 74). Consequently, the ontological and epistemological perspectives on life, humanity, existence, health, moral and suffering embodied and communicated by military chaplains are deemed essential, particularly in conflict and war contexts.

### Areas of distinctive expertise

While the conventional understanding portrays military chaplains and psychiatrists and psychologists as fundamentally distinct categories, this distinction doesn’t universally apply, particularly within the realm of (military) chaplaincy services. As early as the 1930s and 1940s, the development of Clinical Pastoral Education (CPE) in North America, notably in the United States, marked a significant shift (75). This initiative emerged as a response to modernizing hospital contexts, aiming to adapt the roles of priests and pastors to incorporate soul care within healthcare settings (76, 77). During this period, concepts such as pastoral care and counseling were coined (77, 78), blurring the traditional boundaries between pastoral care and psychological support (79). The adoption and spread of Clinical Pastoral Education (CPE) and its associated approaches extended beyond North America to Europe and the Nordic countries, albeit to varying degrees among both pastoral care and counseling and chaplain services (80, 81). Consequently, terminology, language usage, thought processes, and methodologies within chaplaincy have undergone influence and transformation by the psychiatric/psychological paradigm. This integration of psychiatric and psychological theories and practices into chaplains’ work, including within military contexts, reflects a methodological shift in their approach to providing holistic support and care. While the integration of Clinical Pastoral Education (CPE) into the work of some military chaplains represents a significant shift toward a psychiatric paradigm (including psychology), it’s important to recognize that not all chaplains have adopted this approach. Those who have incorporated CPE have not necessarily abandoned their ontological and epistemological perspectives but have instead adopted a more hybrid approach, blending both traditional and modern methodologies to varying degrees (29). As a result, there are instances where military chaplains also function as therapists, demonstrating the fluidity of their roles and responsibilities.

Furthermore, the traditional categorization is challenged by the emergence of secular humanist military chaplains who do not adhere to ontological assumptions of a transcendent dimension, or epistemologies rooted in sacred scriptures, traditions, and practices. These examples underscore the complexity and diversity within military chaplaincy, challenging conventional understandings and highlighting the need for a more nuanced approach to categorization. This subgroup within the category of military chaplains operates within a secular ontology and epistemology, aligning with the foundational assumptions of the psychiatric/psychological paradigm.

### Mixed medical teams

Moreover, instances of close collaboration between military chaplains and other medical professionals, forming mixed medical teams within military hospital settings, have been observed recently. While such collaborations can offer advantages (14), they also pose risks of military chaplains assimilating too closely to a clinical paradigm, potentially diluting the unique contributions stemming from their distinct education, training, experiences, and traditions (1). These dynamics underscore the ongoing tension between integration and preservation of the distinct role and identity of military chaplains within evolving healthcare contexts.

## Unlocking the potential: leveraging military chaplains as a vital resource across deployment phases

Military chaplains are a valuable resource within military organizations, contributing in various ways that complement psychiatrists, psychologists, and others through the chaplains’ specific competencies and expertise. It is advantageous for commanders to have a general understanding of how to unlock this potential. The following key insights can be valuable across three main phases: a) pre-deployment training, b) deployment, and c) post-deployment, which includes homecoming, reintegration into regular military life, or transitioning to civilian life (thereby changing the commander’s formal responsibility to service members).

### Creating conditions for military chaplains to be present across all phases

The role of military chaplains throughout these phases is best understood through the concept of *ministry of presence* (39, 40, 42, 43), as elaborated by Aune et al. (44) and White (45). Thus, limited opportunities for military chaplains to engage in a ministry of presence pose significant challenges to their effectiveness. When chaplains are constrained in their ability to be physically present with military personnel, their capacity to provide support and assistance is compromised. This also applies to their ability to learn the military culture and develop cultural competence. Such a situation can lead to frustration, particularly for chaplains accustomed to actively engaging with service members through a ministry of presence. Moreover, when circumstances dictate that chaplains cannot offer the level of support needed by service members due to specific situational constraints, it can further exacerbate these challenges.

### What military chaplains can do before and during deployment

The phases of pre-deployment training and deployment place extraordinary demands on both military personnel (and their families) and military commanders. During these stages, the primary mission focus is on preparing for and executing the deployment, with the overarching goal of returning home safely. In this environment of intense mission focus, all resources and forces, including military chaplains, converge to support the process and ensure success on the battlefield. Both prior to and during deployment, military chaplains play a crucial role in shaping and reinforcing the moral compass and identity of service members. They have the capacity to nurture the moral imagination (82) and foster an ethical environment within their identities and mindsets, aiding in the establishment and reinforcement of moral and ethical norms that steer the conduct of service members. Furthermore, they offer guidance and assistance in sustaining and adapting this moral compass throughout deployments. It is widely recognized that violations of moral principles or failure to uphold moral character can result in moral conflicts and injuries (4, 7, 62, 83, 84). Whetham (85) argues that it is possible to lose the battle or war you have already won if it is fought unethically. To avoid unethical behavior in morally challenging situations, careful preparation is crucial. Such preparations are important to ready soldiers for the mission and the likely conflicts of values that may arise, helping to prevent moral injury (86). Chaplains can make a valuable contribution to these preparations through their extensive training. They combine their theological, philosophical, and pastoral skills with ethics education to prepare soldiers for the moral challenges of the mission ahead.

### What military chaplains can do during transition

The transition from the deployment context and battlefield back to the routine military life at the home base, known as the post-deployment phase, can present challenges for service members, veterans, families, leaders, and military organizations alike (7, 87–90). During this phase, the readjustment to ordinary military home base activities and life takes precedence over the intense mission focus of deployment, as the urgency of operational demands gradually diminishes. As the mission focus gradually diminishes and other identities and aspects of life gain more prominence, suppressed feelings and thoughts from deployment may resurface among service members and veterans. During this phase, individuals may allow themselves to experience, reflect upon, and ruminate on these emotions in a more profound manner, especially as the influence of the warrior mask or persona on the self begins to wane. This period often signifies a growing need among service members and veterans to address existential, spiritual, religious, and moral concerns, prompting them to seek out conversations with military chaplains for support and guidance (17).

To facilitate the transition from deployment to post-deployment, many armed forces implement Third Location Decompression (TLD) programs (91–93). These TLD initiatives typically adopt a multidisciplinary approach, wherein military chaplains play a crucial role. They contribute through their ministry of presence, attentively listening to participants, and actively engaging by orchestrating meaningful moments of reflection and ceremonies. These activities aid in transitioning service members from a warrior mindset, essential during deployment, to a peace-time military mindset required in the post-deployment phase.

## Unlocking the potential: attending to the moral continuum

Another significant takeaway concerning military chaplains pertains to the concept of the moral continuum.

### Through presence, a unique opportunity arises to grasp and engage with the moral continuum

**Figure 1:**
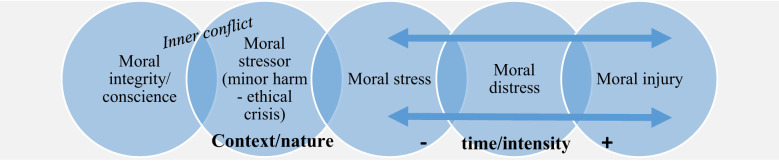
The integrated moral distress and injury scale (including context/nature and time/intensity dimensions of moral stressors and their health-related outcomes).

Recent research, integrating theories of moral distress and moral injury, proposes the existence of a moral continuum, as depicted in the figure below (94) (**Figure 1**). The moral continuum should be considered across all three phases, potentially necessitating slightly different approaches to chaplaincy support.

Considering the moral continuum, it is crucial to address and disrupt negative and potentially destructive moral distress spirals before they escalate into deep-seated moral injuries (94). In the context of military operations, moral stress and distress are often unavoidable due to the necessity, from a civilian perspective, to breach moral and ethical boundaries in order to accomplish combat objectives (3, 6). While some degree of moral stress is inherent in military service due to the conflicting cultural identities of military and civilian life within individuals, it should not necessarily be problematized if killing combatants in accordance with the rules of engagement does not evoke moral discomfort (17). Military culture often imbues this act with positive morals, values, respect, pride, and even recognition through medals (3). However, commanders must recognize the importance of addressing negative moral distress spirals before they escalate too far. For instance, exposure to unjust war events may exert a more substantial and enduring influence on mental health (95). In this regard, military chaplains can serve as invaluable resources and supports.

### Contributing a distinct ontology and epistemology to moral and ethical discourse

While not all military chaplains within NATO may be well-versed in theoretical perspectives and concepts of moral injury, including associated screening instruments and clinically tailored pastoral methods, they possess extensive expertise in navigating moral and ethical dilemmas within a military context. Drawing from diverse faith traditions, backgrounds, and training, chaplains are experienced in addressing issues such as morality, ethics, guilt, shame, suffering, pain, and existential questions. Indeed, fundamental concepts rooted in the religious domain, such as acceptance, forgiveness, reconciliation, atonement, restoration and vindication have been identified as primary pathways to healing and recovery from moral injury (2, 12, 14, 15, 37, 38, 57). Military chaplains are well-equipped to engage in and facilitate conversations surrounding these concepts, providing a safe and confidential space for service members to explore and address moral distress, conflict, and injury. Furthermore, chaplains provide a non-judgmental sanctuary outside the hierarchical structure, where individuals can freely engage in sensitive discussions without fear of career repercussions, thus nurturing trust and openness in such interactions. This aspect can be exceptionally significant, even crucial, for service members, as many may harbor concerns that their thoughts and emotions could be documented and impact their careers and future deployments. However, this is not the case when confiding in military chaplains under the umbrella of absolute secrecy. Unlike other support networks and channels within the military, chaplains operate outside the surveillance apparatus and clinical powers, as delineated by Foucault (71, 72), which are intrinsic to modern medicine. This unique position allows chaplains to provide a confidential and non-judgmental space for service members to express themselves freely without fear of repercussions.

It’s crucial to recognize that military chaplains operate distinctly from psychiatrists or psychologists within the military medical paradigm. Unlike medical professionals who diagnose and implement treatment interventions for diseases, including medications and evidence-based therapies, chaplains approach their work with different mindsets, understandings, assumptions, and premises. Their focus lies primarily on providing spiritual and emotional support, guidance, and chaplaincy care, drawing from their spiritual, religious, and moral frameworks. This distinction does not imply that military chaplains are less effective or inferior in their roles, nor does it suggest that psychiatrists and psychologists would outperform them. Rather, it highlights those military chaplains approach conflicts and issues from alternative ontological and epistemological perspectives. These perspectives are often better suited for addressing complex moral, emotional, and spiritual dilemmas such as guilt, shame, betrayal, suffering, pain, and existential issues (12).

## Unlocking the potential: addressing death

Furthermore, military chaplains specialize in the domain of death, regularly addressing and working with death and grief within the framework of their diverse faith traditions. This expertise is invaluable across all three phases mentioned earlier, with each phase accentuating slightly different facets of chaplaincy support. At the commanding level, it is equally imperative to plan, integrate, and train military chaplains and units for potential mass death and mass casualty outcomes in military conflicts and operations. This proactive approach ensures the establishment of effective chaplaincy routines and logistical frameworks, enabling the resolution of such future scenarios in a morally dignified manner (see, for example, 96). During pre-deployment and deployment, the focus is on equipping military units with the necessary abilities and resources to address death and grief in a dignified, established, and culturally accepted manner. This is essential for fostering optimal conditions to sustain task execution, maintain mission focus, and achieve victory in terms of morale, combat effectiveness, and strategic objectives.

In the post-deployment phase, addressing death and grief stemming from deployments may require renewed attention and may involve both traditional and innovative approaches, such as rituals and grief work (17). Military chaplains, renowned for their expertise and extensive experience in handling death and grief, are readily available to commanders for support and guidance during this critical phase.

## Conclusion

This collaborative inquiry between researchers in military chaplaincy and military chaplains in HMF-RTG-352 aimed to enhance the understanding of military chaplains within NATO by elucidating key aspects of their practice, highlighting what sets them apart, and providing general recommendations for how military commanders at the tactical (battalion) level can benefit from chaplains during all phases of deployment—pre-deployment, deployment, and post-deployment.

The results of this inquiry should be seen as a preliminary, non-exhaustive step in a process aimed at improving the ability to address moral challenges in the future security environment, including those faced by military chaplains. It is reasonable to assume that military personnel and commanders within NATO will face moral conflicts between right and wrong, existential and spiritual challenges (e.g., questions about life, purpose, and meaning), including the reality of death. They may need the ability to receive and utilize support from military chaplains when their own or other chaplains are absent, insufficient, injured, or have fallen. The International Military Chief of Chaplains Conference (IMCCC) has established minimal guidelines for chaplain cooperation in a multinational environment and continues to work towards greater interoperability (97).

Presently, there is a burgeoning interest in military chaplaincy services within NATO, evidenced by the presence of chaplains in NATO’s Science and Technology Organization Human Factor and Medicine’s Research Task Groups and the initiation of an HFM Exploratory Team 209. Throughout 2023-2024, this exploratory team^2^ delved into the spiritual dimension within military medicine, emphasizing the indispensable contribution of military chaplains. Their efforts led to the proposal of a new Research Task Group, HFM-408, titled “Chaplains and Spiritual-Existential Wellbeing,” which will systematically explore the topic further. Additionally, a workshop on the “Spiritual Dimension of Military Health and Resilience” has been approved and is scheduled to take place in Stockholm in October 2025. These NATO initiatives strive to develop and deepen the questions that this article has begun to explore. There is still considerable work to be done in this area, both in terms of research and practice, particularly regarding interoperability and the common ground that various and diverse military chaplains can agree upon.

Lastly, a limitation of this article is that military chaplains in each NATO country undertake diverse tasks specific to their country, culture, societal and military laws, and armed forces, including various branches. Given the diversity within the NATO context, identifying generic patterns regarding military chaplains is a significant challenge in itself. While detailing the unique features of military chaplaincy services within each NATO country is beyond the scope of this article, such nuanced insights are best explored through dedicated research teams, projects, and programs within each nation, or by seeking detailed information from their chief of chaplains, appointed representatives, or the nearest military chaplain.

## Data Availability

The original contributions presented in the study are included in the article/supplementary material, further inquiries can be directed to the corresponding author/s.

## Footnotes

1 All authors were also appointed members of HFM-ET-209 on the spiritual dimension of military medicine. For a description of HMF-ET-209 see https://www.sto.nato.int/search/Pages/activities_results.aspx?k=ET%20209ands=Search%20Activities

2 All authors were appointed members of HFM-ET-209, including chairs and co-chairs.
